# Weighted Blankets for Agitation in Hospitalized Patients with Dementia: Protocol for a Randomized Controlled Trial

**DOI:** 10.2196/57264

**Published:** 2025-02-26

**Authors:** Holly A Schenzel, Allyson K Palmer, Neel B Shah, Donna K Lawson, Karen M Fischer, Maria I Lapid, Ruth E DeFoster

**Affiliations:** 1 Division of Hospital Internal Medicine Mayo Clinic Rochester, MN United States; 2 Robert and Arlene Kogod Center on Aging Mayo Clinic Rochester, MN United States; 3 Department of Quantitative Health Sciences Mayo Clinic Rochester, MN United States; 4 Department of Psychiatry and Psychology Mayo Clinic Rochester, MN United States

**Keywords:** dementia, hospitalized dementia patients, agitation, aggression, behaviors, sleep, weighted blankets, nonpharmacologic strategy, pilot study, inpatients, occupational therapy

## Abstract

**Background:**

There are limited therapies approved for the treatment of aggression and agitation in patients with dementia. While antipsychotics and benzodiazepines are commonly used, these medications have been associated with significant side effects and US Food and Drug Administration (FDA) boxed warnings. Weighted blankets have been associated with decreased anxiety and improved sleep. Weighted blankets are potentially a nonpharmacologic option to reduce agitation in hospitalized patients with dementia.

**Objective:**

The aim of this study is to investigate the effect of weighted blankets on aggression and agitation in hospitalized patients with dementia.

**Methods:**

A pilot study will be conducted on a total of 30 hospitalized patients with a documented clinical diagnosis of dementia and ongoing agitated behaviors admitted to a medicine or psychiatry service. Patients will be randomly allocated to receive either a weighted blanket for 3 nights or continued usual care. The primary outcome is the change in the observational version of the Cohen-Mansfield Agitation Inventory (CMAI-O) over the course of the 3-night study period. The secondary outcomes are changes in Edmonton Symptom Assessment System Revised (ESAS-r) and Clinical Global Impression (CGI) scores, hours of sleep, use of antipsychotics and benzodiazepines, and incidence of delirium. Identical study assessments will be completed for both the usual care and the weighted blanket study groups. At 5 study time points (baseline, postnight 1, postnight 2, postnight 3, and a final assessment 48-72 h after the last use of the weighted blanket), patients will be assessed with the CMAI-O, ESAS-r, and CGI tools. All assessments will be completed by the bedside nurse or patient care assistant caring for the patient each day. Within 2 to 4 weeks post discharge from the hospital, study coordinators will contact the patient’s legally authorized representative (LAR) to assess for continued use of the weighted blanket.

**Results:**

Enrollment of participants began on April 23, 2023. As of November 2024, a total of 24 participants have been enrolled in the study. Baseline characteristics of enrolled participants will be analyzed and reported upon completion of enrollment. We anticipate completing data collection by March 2026.

**Conclusions:**

The study will determine the effect of weighted blankets on agitation in hospitalized patients with dementia. Insights into the effect of weighted blankets on sleep will also be gained. The results of this study will be relevant in the setting of increasing numbers of older adults with dementia exhibiting agitation, leading to increased hospitalizations, caregiver burden, and health care costs.

**Trial Registration:**

ClinicalTrials.gov NCT03643991; http://clinicaltrials.gov/ct2/show/NCT03643991

**International Registered Report Identifier (IRRID):**

DERR1-10.2196/57264

## Introduction

### Background

Many patients with dementia develop neuropsychiatric symptoms and somewhere between 40% and 76% of patients demonstrate agitation and aggression [1,2]. Agitated behaviors are often disruptive and difficult to manage, and are associated with increased caregiver burden and burnout, increased hospitalizations, and elevated health care costs [1,3]. Agitation includes a range of behaviors, including restlessness, pacing, physical aggression, and verbal agitation [4].

Nonpharmacologic strategies are the first line in the prevention and treatment of neuropsychiatric symptoms of dementia, including agitation. Limited pharmacologic options exist in this setting. In fact, no pharmacologic therapies were approved by the Food and Drug Administration (FDA) for this indication until the recent approval of the atypical antipsychotic brexpiprazole for the treatment of agitation associated with dementia due to Alzheimer disease [5]. Other antipsychotics are commonly used, particularly in emergency and acute care settings when agitated or aggressive behaviors threaten either patient or caregiver [6]. These medications, however, are associated with significant serious long- and short-term side effects including akathisia and restlessness [7], and carry an FDA-boxed warning for increased risk of mortality [8]. The 2016 American Psychiatric Association practice guidelines recommend that antipsychotic medications be reserved for severe, dangerous, or significantly distressing symptoms and used at the lowest effective dose due to concern for side effects [9]. The management of agitated and aggressive symptoms without primary reliance on antipsychotics remains a challenge. Benzodiazepines are typically avoided due to the risk of cognitive impairment, falls, and respiratory depression [10].

Weighted blankets are filled with plastic pellets or beads and are commercially available in weights ranging from 5 to 30 pounds. Weighted blankets have been used in occupational therapy as a form of noninvasive deep-pressure stimulation, which appears to increase parasympathetic arousal and decrease anxiety [11-13]. Weighted blankets have been studied in patients with psychiatric diagnoses in both the inpatient and outpatient settings, and they appear to be associated with decreased anxiety and improved sleep [14,15]. Weighted blankets have also been associated with improved chronic pain and reduced anger scores [16,17]. Currently, however, there are no studies investigating the use of weighted blankets to address agitation in hospitalized patients with dementia.

### Objectives

The aim of this study is to investigate the effect of weighted blankets on aggression and agitation in hospitalized patients with dementia. We hypothesize that compared to controls, patients with agitation in the setting of dementia will show reduced agitation, reduced anxiety, and potentially improved sleep while hospitalized when provided with a weighted blanket.

## Methods

### Design and Randomization

To address this question, we designed a pilot study (ClinicalTrials.gov NCT03643991) with 1:1 randomization of hospitalized patients with dementia and evidence of agitated behaviors to receive either a weighted blanket for 3 nights or continued usual care.

#### Study Population and Recruitment

Patients will be recruited from a single large tertiary care hospital. Participants are eligible for inclusion if they are 60 years or older, have a documented clinical diagnosis of dementia, and are currently admitted to either a medicine or psychiatry primary service. All participants are required to have ongoing agitated behaviors at the time of enrollment as demonstrated by an elevated CMAI-O with a score of 2, 3, or 4 on at least one aggression-related item (items 1-11 or 22-24) [18]. Patients will be excluded if they have severe pain likely to be exacerbated by the use of the weighted blanket, have significant burns or wounds for which weighted blanket use could potentially be detrimental, or are admitted on a 72-hour involuntary hold. Participants can be enrolled at any point during their hospitalization. Hospitalizations will not be prolonged for the purpose of the study. To reflect real-world conditions, all other usual medical care will be continued at the discretion of the primary medical or psychiatric team.

#### Intervention

Participants will be randomized 1:1 to either the usual care group or the weighted blanket group. The weighted blankets used are commercially available and purchased for the study. Patients randomized to the weighted blanket group will be provided with either a 10 lb, 15 lb, or 20 lb weighted blanket, ensuring that the weight is not more than 10% of the participant’s body weight. Participants will be withdrawn from the study if they refuse the blanket or are unable to tolerate it for more than 2 hours on the first night after enrollment. Participants can also be withdrawn from the study at any time if the primary team feels that further participation will jeopardize patient safety. Enrolled participants will use the blanket for a total of 3 consecutive nights, or until the time of discharge if that occurs sooner. Following participation in the study, participants will be allowed to keep the weighted blanket for future personal use. To reduce potential bias, the data analysis team will remain blinded to group assignment during statistical analyses, although investigators administering the questionnaires will not be blinded due to the nature of the intervention.

#### Primary Outcome

The primary end point will be the change in CMAI-O over the course of the 3-night study period. The CMAI-O is a validated scale published in 2020 by Griffith et al [18] to quantify neuropsychiatric behavior in patients with dementia. Observers rank the frequency of 29 behaviors grouped into categories: physical aggressive behaviors (questions 1-11), physical nonaggressive behaviors (questions 12-21), verbal aggressive behaviors (questions 22-24), and verbal nonaggressive behaviors (questions 25-29). The frequency of each behavior is ranked as 1 (never), 2 (less than once per h), 3 (once per h), and 4 (several times per hour).

#### Secondary Outcomes

Secondary end points will include change in Edmonton Symptom Assessment System Revised (ESAS-r) score and change in Clinical Global Impression (CGI) score as well as hours of sleep, use of antipsychotics and benzodiazepines, and incidence of delirium.

The ESAS-r was originally designed as a self-report tool in which patients rate the severity of 9 symptoms on a scale from 0 to 10 (10 being the most severe) [19]. The tool assesses the following nine symptoms: pain, tiredness, drowsiness, nausea lack of appetite, shortness of breath, depression, anxiety, and well-being. This assessment tool has been commonly used in palliative medicine and oncology settings, translated into several languages, and is well received by patients and nursing staff [19]. The ESAS-r captures the pattern of a patient’s severity of symptoms at a point in time and may be repeated to track changes over time. Since patients with a diagnosis of dementia vary significantly in their ability to report symptoms, we have standardized the protocol by using caregiver (nurse or patient care assistant) input to complete the ESAS-r assessment tool [19].

The CGI scale is a brief, independent assessment initially designed to summarize a caregiver’s view of a patient’s global functioning before and after initiating an intervention [20]. The CGI consists of 2 one-item measures that evaluate the severity of psychopathology and change from the initiation of treatment on a 7-point scale. The CGI may be used to track clinical progress over time [20].

Further clinical data will be extracted from the medical record. Caregiver staff will estimate hours of sleep. Use of antipsychotics and benzodiazepines will be recorded based on the medical administration records. The incidence of delirium will be estimated based on documentation of a newly positive Brief Confusion Assessment Model (bCAM) assessment [21]. Delirium Triage Screen and bCAM assessments are performed to screen for delirium every 12 hours (once per nursing shift) in the hospital where this study will be conducted.

#### Study Timeline and Follow-Up

Identical study assessments will be completed by the patient’s caregiver (either registered nurse or patient care assistant) for both the usual care group and the weighted blanket study group. Cohen-Mansfield Agitation Inventory (CMAI-O), ESAS-r, and CGI scores will be collected at 5 study time points (baseline, postnight 1, postnight 2, postnight 3, and a final assessment 48-72 hours after the last use of the weighted blanket). A total of 3 nights were determined to be sufficient for assessing trends in agitation, anxiety, and sleep, while also testing the feasibility of the intervention in a real-world clinical environment. Within 2-4 weeks following hospital discharge, study coordinators will contact the patient’s legally authorized representative (LAR) to assess for continued use of the blanket. Refer to the patient timeline (Table 1).

**Table 1:** Patient timeline.

Assessment	Baseline (enrollment)	WB^a^ day 1	WB day 2	WB day 3	Final within 72 hours of last use of WB (cohort 1) or no WB (cohort 2)	Within 2-4 weeks post discharge
CMAI-O^b^	✓	✓	✓	✓	✓	
ESAS-r^c^	✓	✓	✓	✓	✓	
CGI^d^	✓	✓	✓	✓	✓	
Posthospitalization phone call to assess continued weighted blanket use						✓

^a^WB: weighted blanket.

^b^CMAI-O: Cohen-Mansfield Agitation Inventory.

^c^ESAS-r: Edmonton Symptom Assessment System Revised.

^d^CGI: Clinical Global Impression.

#### Sample Size

We will target an enrollment of 30 participants in this pilot study. This sample size was chosen to balance the need for gathering preliminary data with the constraints of patient availability, time, and resources within our hospital setting. The sample size of 30 is sufficient to provide preliminary insights into the effects of weighted blankets on agitation, allowing us to explore trends and estimate effect sizes for future studies. Recruitment will be facilitated by daily review of hospital-wide behavior emergency response team activations and patient referrals from inpatient medicine and psychiatry primary services.

### Data Collection, Management, and Confidentiality

Participants will be randomized 1:1 to the treatment and usual care groups via computer-generated sequence which will be concealed until after each patient’s enrollment. Given the nature of the study, blinding of caregivers and study coordinators to treatment groups is not feasible.

Data will be collected by study personnel in a manner consistent with patient confidentiality. For patients withdrawn from the study, no further data will be collected but existing study data to the point of withdrawal will be included in the final analysis which will be performed based on intention to treat.

All data obtained will be entered into REDCap (Research Electronic Data Capture), a secure electronic database that is password-protected and accessible only to the study team. Each participant will be assigned a unique identification code to protect their identity, and only the code will be used for forms, reports, and data analysis. Paper records containing protected health information will be maintained in locked file cabinets in a secure room accessible only to research personnel. All patient information will be kept confidential and managed according to the Health Insurance Portability and Accountability Act of 1996 requirements.

### Data Analysis

A per-protocol analysis will be used. Baseline characteristics of participants will be reported using percentages for categorical variables and mean with SD for continuous variables. Differences between the 2 treatment groups for the descriptive variables will be compared using chi-square tests for categorical variables and Kruskal-Wallis tests for continuous variables. The medications and dosages taken during the study will also be reported. The primary outcome of the CMAI-O score will be described at each of the 5 time points (baseline, day 1, day 2, day 3, and day 72 follow-up). A linear mixed effects model with time as a random effect will be used to analyze changes in the CMAI-O score over time. Both the unadjusted and an adjusted model with age, sex, and unit type as covariates will be reported. Age and sex were included as covariates as they are standard demographic factors that can influence behavioral outcomes. Unit type (medicine vs psychiatry) was included as it may impact patient management practices and potentially influence study outcomes. This model also allows for missing data which occurs in the dataset when a patient was discharged before the end of the study period. Secondary outcomes of ESAS-r and CGI scores will be analyzed in a comparable way with a linear mixed model. The end-of-study survey results will be described for each question. A per-protocol analysis will evaluate only the enrolled patients who followed protocol.

### Monitoring

The principal investigator will conduct weekly meetings with the study coordinator and research team to review the status of the study, recruitment and enrollment of participants, protocol adherence, safety issues, and overall conduct of the study. The principal investigator will monitor, track, and report any adverse events, protocol deviations, and progress reports or continuing reviews to the institutional review board (IRB) with research regulations.

### Ethical Considerations

This study follows the SPIRIT (Standard Protocol Items: Recommendations for Interventional Trials) guidelines for reporting (see Multimedia Appendix 1). The study protocol was approved by the Mayo Clinic IRB (IRB 17-009951) and was registered with ClinicalTrials.gov (NCT03643991). Amendments to the protocol will be submitted to the Mayo Clinic IRB for approval before implementation. Written informed consent approved by the IRB will be obtained from the participant’s LAR, who will be provided sufficient information regarding the risks and benefits of the study to make an informed decision about the participation of their loved ones in the study. The LARs will be fully informed about their right to withdraw participation at any time. No compensation is being provided to participants, although individuals randomized to the weighted blanket group are permitted to keep the weighted blanket. Study investigators will have access to the final dataset, which will include deidentified participant information only as outlined above in the “Data Collection, Management, and Confidentiality” section. Results will be submitted to a peer-reviewed journal and scientific conferences for the advancement of clinical care.

## Results

Enrollment of participants began on April 23, 2023. As of November 2024, 24 participants have been enrolled in the study. Baseline characteristics of enrolled participants will be analyzed and reported upon completion of enrollment. We anticipate completing data collection by March 2026. The study results will be reported in a peer-reviewed journal upon completion.

## Discussion

### Primary and Secondary Outcomes

We hypothesize the use of a weighted blanket will reduce patients’ behaviors and agitation; therefore, patients’ CMAI-O scores will decrease. In addition, we anticipate patients’ ESAS-r and CGI scores will decrease with the use of a weighted blanket. We hope with reduced behaviors and agitation that patients will have reduced antipsychotic and benzodiazepine administrations and decreased incidence of delirium.

### Strengths and Limitations

There are several strengths of this study. This study is a randomized controlled trial being performed in both medical and psychiatric inpatient settings at an academic medical center. The study’s intervention is a nonpharmacologic option that has minimal potential side effects. The study is a pilot study that can offer opportunities for further research studies. A couple of the study’s limitations are the small sample size and performed at a single organization. In addition, the investigators are nonblinded and could lead to a potential bias. It is unclear if the results will be generalizable to all patients with agitation and dementia, other health care organizations, and outpatient settings.

### Conclusions

This study addresses the important clinical issue of agitation in hospitalized patients with dementia. This study will contribute to the growing body of knowledge on nonpharmacological management of behavioral symptoms in older adults with dementia. This is important as the management of agitated and aggressive symptoms without primary reliance on antipsychotics and benzodiazepines remains a challenge. We anticipate that these study results will be used to inform health care professionals, caregivers, and family members regarding the potential usefulness of weighted blankets as a nonpharmacologic method to decrease agitation and distress in hospitalized older adults with dementia.

## Appendix

Multimedia Appendix 1 SPIRIT Outcomes 2022 checklist.

## Abbreviations

bCAM: Brief Confusion Assessment Model
CGI: Clinical Global Impression
CMAI-O: Cohen-Mansfield Agitation Inventory
ESAS-r: Edmonton Symptom Assessment System Revised
FDA: US Food and Drug Administration
IRB: institutional review board
LAR: legally authorized representative
REDCap: Research Electronic Data Capture
SPIRIT: Standard Protocol Items: Recommendations for Interventional Trials

## Data Availability

The datasets generated or analyzed during this study are available from the corresponding author on reasonable request.
